# Declining responsiveness of childhood *Plasmodium falciparum* infections to artemisinin-based combination treatments ten years following deployment as first-line antimalarials in Nigeria

**DOI:** 10.1186/s40249-019-0577-x

**Published:** 2019-08-06

**Authors:** Akintunde Sowunmi, Godwin Ntadom, Kazeem Akano, Folasade O. Ibironke, Adejumoke I. Ayede, Chimere Agomo, Onikepe A. Folarin, Grace O. Gbotosho, Christian Happi, Stephen Oguche, Henrietta U. Okafor, Martin Meremikwu, Philip Agomo, William Ogala, Ismaila Watila, Olugbenga Mokuolu, Finomo Finomo, Joy C. Ebenebe, Nma Jiya, Jose Ambe, Robinson Wammanda, George Emechebe, Wellington Oyibo, Francis Useh, Temitope Aderoyeje, Titilope M. Dokunmu, Omobolaji T. Alebiosu, Sikiru Amoo, Oluwabunmi K. Basorun, Olubunmi A. Wewe, Chukwuebuka Okafor, Odafe Akpoborie, Bayo Fatunmbi, Elsie O. Adewoye, Nnenna M. Ezeigwe, Ayoade Oduola

**Affiliations:** 1 Antimalarial Therapeutic Efficacy Monitoring Group, National Malaria Elimination Programme, The Federal Ministry of Health, Abuja, Nigeria; 2 Department of Pharmacology and Therapeutics, University of Ibadan, Ibadan, Nigeria; 3 Institute for Medical Research and Training, University of Ibadan, Ibadan, Nigeria; 4 Department of Clinical Pharmacology, University College Hospital, Ibadan, Ibadan, Nigeria; 5 Department of Biological Sciences and African Centre of Excellence for Genomics of Infectious Diseases (ACEGID), Redeemer University, Ede, Nigeria; 6 Department of Paediatrics, University of Ibadan, Ibadan, Nigeria; 7 Department of Medical Laboratory Science, University of Lagos, Lagos, Nigeria; 8 Department of Pharmacology and Toxicology, University of Ibadan, Ibadan, Nigeria; 9 Department of Paediatrics, University of Jos, Jos, Nigeria; 10 Department of Pediatrics, Institute of Child Health, University of Nigeria Teaching Hospital, Enugu, Nigeria; 11 Department of Paediatrics, University of Calabar, Calabar, Cross River State Nigeria; 12 Nigeria Institute of Medical Research, Lagos, Nigeria; 13 Department of Paediatrics, Ahmadu Bello University, Zaria, Nigeria; 14 Department of Paediatrics, Specialist Hospital, Maiduguri, Nigeria; 15 Department of Paediatrics and Child Health, University of Ilorin, Ilorin, Nigeria; 16 Department of Paediatrics, Federal Medical Centre, Yenagoa, Nigeria; 17 Department of Paediatrics, Nnamdi Azikiwe University, Awka, Nigeria; 18 Department of Paediatrics, Uthman Dan Fodio University, Sokoto, Nigeria; 19 Department of Paediatrics, University of Maiduguri, Maiduguri, Nigeria; 20 Department of Paediatrics, Imo State University Teaching Hospital, Orlu, Nigeria; 21 Department of Medical Microbiology and Parasitology, University of Lagos, Lagos, Nigeria; 22 Department of Medical Laboratory Science, University of Calabar, Calabar, Nigeria; 23 Department of Biochemistry, Covenant University, Ota, Nigeria; 24 World Health Organization, Country Office, Kampala, Uganda; 25 Department of Physiology, University of Ibadan, Ibadan, Nigeria; 26 University of Ibadan Research Foundation, University of Ibadan, Ibadan, Nigeria

**Keywords:** Declining responsiveness, Falciparum malaria, Children, Artemisinin-based combination treatment, Nigeria

## Abstract

**Background:**

The development and spread of artemisinin-resistant *Plasmodium falciparum* malaria in Greater Mekong Subregion has created impetus for continuing global monitoring of efficacy of artemisinin-based combination therapies (ACTs). This post analyses is aimed to evaluate changes in early treatment response markers 10 years after the adoption of ACTs as first-line treatments of uncomplicated falciparum malaria in Nigeria.

**Methods:**

At 14 sentinel sites in six geographical areas of Nigeria, we evaluated treatment responses in 1341 children under 5 years and in additional 360 children under 16 years with uncomplicated malaria enrolled in randomized trials of artemether-lumefantrine versus artesunate-amodiaquine at 5-year interval in 2009–2010 and 2014–2015 and at 2-year interval in 2009–2010 and 2012–2015, respectively after deployment in 2005.

**Results:**

Asexual parasite positivity 1 day after treatment initiation (APPD1) rose from 54 to 62% and 2 days after treatment initiation from 5 to 26% in 2009–2010 to 2014–2015 (*P* = 0.002 and *P* <  0.0001, respectively). Parasite clearance time increased significantly from 1.6 days (95% confidence interval [*CI*]: 1.55–1.64) to 1.9 days (95% *CI*, 1.9–2.0) and geometric mean parasite reduction ratio 2 days after treatment initiation decreased significantly from 11 000 to 4700 within the same time period (*P* <  0.0001 for each). Enrolment parasitaemia > 75 000 μl^− 1^, haematocrit > 27% 1 day post-treatment initiation, treatment with artemether-lumefantrine and enrolment in 2014–2015 independently predicted APPD1. In parallel, Kaplan-Meier estimated risk of recurrent infections by day 28 rose from 8 to 14% (*P* = 0.005) and from 9 to 15% (*P* = 0.02) with artemether-lumefantrine and artesunate-amodiaquine, respectively. Mean asexual parasitaemia half-life increased significantly from 1.1 h to 1.3 h within 2 years (*P* <  0.0001).

**Conclusions:**

These data indicate declining parasitological responses through time to the two ACTs may be due to emergence of parasites with reduced susceptibility or decrease in immunity to the infections in these children.

**Trial registration:**

Pan African Clinical Trial Registration PACTR201508001188143, 3 July 2015; PACTR201508001191898, 7 July 2015 and PACTR201508001193368, 8 July 2015 PACTR201510001189370, 3 July 2015; PACTR201709002064150, 1 March 2017; https://www.pactr.samrca.ac.za

**Electronic supplementary material:**

The online version of this article (10.1186/s40249-019-0577-x) contains supplementary material, which is available to authorized users.

## Multilingual abstracts

Please see Additional file 1 for translations of the abstract into the five official working languages of the United Nations.

## Background

The emergence and spread of resistance in *Plasmodium falciparum* to artemisinin in the Greater Mekong Subregion (GMS) [1–7] threatens the treatment and control of *P. falciparum* malaria globally [8]. Although in Africa, there is currently no evidence of resistance in *P. falciparum* to artemisinin in indigenous population [9–11], declining responsiveness manifested as increasing proportion of patients with residual asexual parasitaemia (asexual parasite positivity) 1 day after treatment initiation (APPD1) and apparent increases in rate of recrudescent infections following artemisinin-based combination therapies (ACTs) has been reported from an area of seasonal intense transmission on the coast of Kenya [12].

One of the measures of declining efficacy to artemisinin-like drugs is a terminal elimination half-time of parasitaemia ≥5 h [8]. In Africa where transmission is high and the burden of malaria is greatest, there is scant data on terminal elimination half-time of parasitaemia following ACTs [9]. In addition, most reported studies on the estimation of terminal elimination half-time of asexual parasitaemia have employed parasite clearance estimator [8, 9] instead of kinetic models which required intense blood sampling.

It is over a decade many African countries adopted and deployed ACTs as first-line treatments of uncomplicated falciparum malaria [13]. In Nigeria, ACTs were adopted and deployed in 2005 [14]. Despite a relatively long period of adoption and deployment, there are few reported periodic country-wide evaluations of the efficacy of ACTs on the African continent [11]. Using data from two country-wide, open-label, randomized efficacy trials at 14 sentinel sites located in six geographical areas of Nigeria over a 5-year interval (2009–2010 and 2014–2015), and at one of the sentinel sites over a 2-year interval (2009–2010 and 2012–2015) [15, 16], we performed a post hoc analyses over time of the in vivo responses to two ACTs, namely: artemether-lumefantrine (AL) and artesunate-amodiaquine (AA) to determine if there was declining responsiveness of childhood *P. falciparum* infections over time following adoption and deployment of the two ACTs as first-line therapies. The studies coincided with five and ten years of almost exclusive first-line treatments with ACTs for all cases of uncomplicated falciparum malaria in Nigeria.

The main objectives of our post hoc analyses are: (1) to determine if there are significant differences in early response markers (for examples, APPD1, residual asexual parasitaemia [asexual parasite positivity] 2 days after treatment initiation [APPD2], parasite reduction ratio 1 or 2 days after treatment initiation [PRRD1 or PRRD2]) and the factors contributing to these significant differences during a five-year study interval; (2) to estimate the terminal elimination half-time of asexual parasitaemia in a subpopulation of children after initiation of ACTs after a 2 year interval of almost exclusive use of ACTs and (3) to determine if there are significant differences in the terminal elimination half-time of asexual parasitaemia following a short period of use of ACTs at one of the sentinel sites.

## Methods

### Study locations

**Fig. 1:**
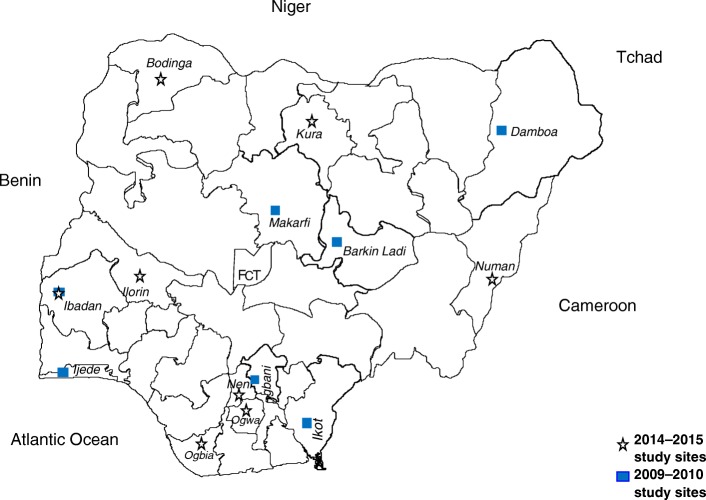
Map of Nigeria showing study sites

The initial studies were conducted between 2009 and 2010 (the end of the first 5 years of deployment) and between 2014 and 2015 (the end of the second 5 years of deployment). They were nested in a National Malaria Elimination Programme for monitoring the therapeutic efficacies of antimalarial drugs at 14 sentinel sites located in six geographical areas of Nigeria, namely: Agbani, Ikot Ansa, Barkin Ladi, Damboa, Ijede, Sabo quarters of Ibadan and Makarfi in Enugu, Cross River, Plateau, Borno, Lagos, Oyo, and Kaduna States, respectively in 2009–2010; and in Ogbia (Otuasegha), Neni, Ogwa, Numan, Ilorin, Kura, Bodinga and Ibadan in Bayelsa, Anambra, Imo, Adamawa, Kwara, Kano, Sokoto and Oyo States, respectively in 2014–2015 (Fig. 1). At virtually all of the sentinel sites, malaria transmission occurs all year round; however, it is more intense during the rainy season from April to October.

### Design of post hoc analyses

**Fig. 2:**
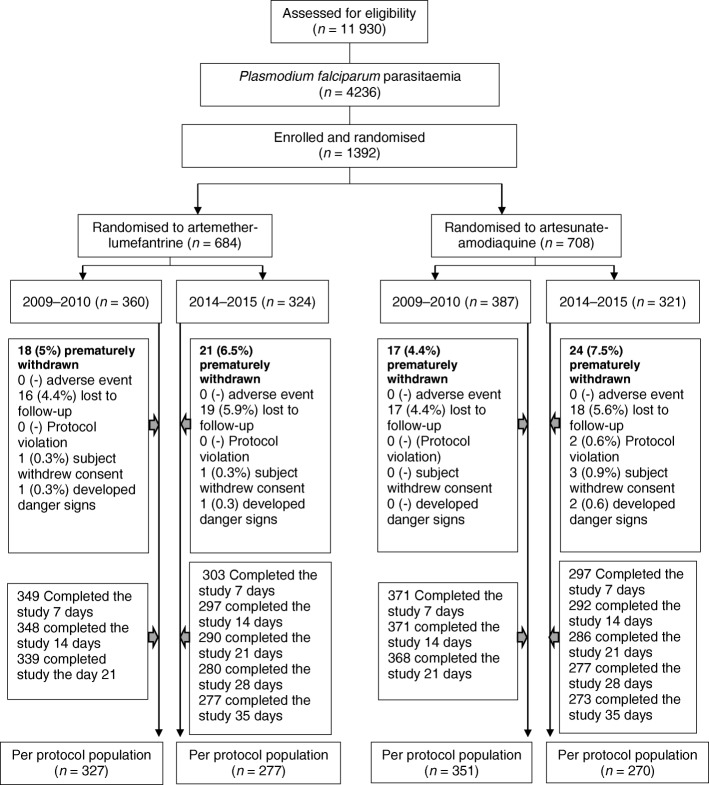
Study profile of children randomised in efficacy study

This is a detailed evaluation of treatment responses at two time periods in open label, randomized trials conducted to monitor the efficacies of AL and AA in under 5-year-old Nigerian children with acute, symptomatic apparently uncomplicated falciparum malaria (Clinical Trial Registration Numbers PACTR201510001189370 and PACTR201709002064150). The primary efficacy endpoints were complete clearance of initial asexual parasitaemia and 28-day cure rate adjusted for re-infection by polymerase chain reaction (PCR) in 2009–2010 [15] but extended to 42 days in 2014–2015 [16]. The secondary endpoints were residual asexual parasitaemia one, two and three days after treatment initiation, parasite reduction ratios one or two days after treatment initiation, fever clearance, and gametocyte carriage. Assuming a cure rate of 100 and 95% with AL and AA, respectively, and a 5% drop out rate, we estimated a minimum of 50 patients per treatment arm in each sentinel site would provide 95% power and a 95% confidence interval. Overall, a total of 1341 children (*n* = 743 and 598 in 2009–2010 and 2014–2015, respectively) were evaluated in the post hoc analyses (Fig. 2).

#### Enrolment of patients

Standardized procedures and protocol were used at all sites. Briefly, patients were eligible for enrollment in the study if they were: aged 6–59 months, had symptoms compatible with acute uncomplicated malaria such as fever, anorexia, vomiting or abdominal discomfort with or without diarrhea with *P. falciparum* mono-infections from ≥1000 μl^− 1^ to 200 000 μl^− 1^ of blood, a body (axillary) temperature > 37.4 °C or in the absence of measured fever, a recent history of fever in the 24 to 48 h before presentation, absence of other concomitant illness, no history of antimalarial drug ingestion in the 2 weeks prior to enrolment, no evidence of severe malaria [17, 18], and parents or guardians gave written informed consent. Using computer generated numbers, patients were allotted to AL or AA treatment groups prior to the commencement of recruitment into the study by personnel who was not involved in the selection and enrolment procedures. Sealed opaque envelopes containing the randomization were only opened by the attending physician at the time of recruitment. Treatment randomization was 1:1 for all patients at all sentinel sites during the two study periods.

### Study drug and administration

Patients were administered with 3 days standard treatment of AL or AA as previously described [15, 16, 19]. Briefly, AL (Coartem®, Novatis, Basel, Switzerland) was given as follows: patients weighing 5–14 kg received one tablet, and those weighing > 14–24 kg received two tablets at presentation (0 h), 8 hours later and at 24, 36, 48 and 60 h after the first dose (each tablet of AL contains 20 mg of artemether and 120 mg of lumefantrine) while AA (Winthrop®, Sanofi Aventis, France) was given as follows: patients weighing > 4.5 to < 9 kg received one tablet, those weighing > 9 to < 18 kg received one tablet and those weighing > 18 to < 24 kg received one tablet of the following formulations: 25 mg/67.5 mg, 50 mg/135 mg, 100 mg/270 mg of fixed dose combination of artesunate/amodiaquine, respectively daily for 3 days. All drugs were given orally. In children who were not able to swallow whole tablets, the tablets were carefully crushed using a tablet crusher, dissolved in water and administered orally. The three daily doses of AA, and that of AL at 0 (first dose at enrolment), eight, 24 and 48 h were given by direct observed therapy (DOT). The second daily doses of AL at 36 and 60 h after the first dose were given by guardians or parents of the children at home. A phone call was made to remind guardians/parents of time of the second daily doses of AL and to monitor the outcome of drug administration. A full replacement of dose was administered if vomiting occurred within 30 min of administration. Patients who vomited the dose after a repeated administration were excluded from the study.

### Study flow and clinical procedures

The day of presentation (day of starting treatment) was regarded as day 0. Thick and thin blood films, taken from a finger prick, were obtained from each child as soon as they came to the clinic and the slides were carefully labelled with the patients’ codes and air-dried before being Giemsa-stained. Follow-up with clinical, parasitological and haematocrit evaluation was done daily on days 1–3 and 7, and thereafter, weekly for additional 3–5 weeks. If symptoms or parasitaemia was present on day 3, patients were also seen for assessment on day 4. Anaemia was defined as haematocrit < 30% and was classified as mild, moderate or severe if haematocrit value was 21–29, 15–20 or < 15%, respectively.

### Parasitological assessment

Parasitaemia, in Giesma-stained thick blood films, was estimated by counting asexual parasites relative to 500 leukocytes, or 500 asexual forms whichever occurred first. From this figure, the parasite density was calculated assuming a leukocyte count of 6000 μl^− 1^ of blood [20]. Presence of sexual forms was noted on blood slides but their densities were not quantified. A slide was considered asexual or sexual parasite negative if no parasite was detected after examination of 200 microscope fields. Asexual parasite reduction ratio (PRR) 1 or 2 days after treatment initiation (PRRD1 or PRRD2), and asexual parasite positivity 3 days after treatment initiation (APPD3), and parasite clearance time (PCT) determined by microscopy or estimation of haematocrit were defined or determined as previously described [16, 21]. Briefly, PRRD1 or PRRD2 was defined as the ratio of day 0/day 1 parasitaemia [that is, ${PRR}_{D1}=\frac{Parasitaemia\  on\  day\ 0}{Parasitaemia\  on\  day\ 1}$] or ratio of day 0/day 2 parasitaemia [that is, ${PRR}_{D2}=\frac{Parasitaemia\  on\  day\ 0}{Parasitaemia\  on\  day\ 2}$], respectively; APPD1–3 as proportion of patients with residual parasitaemia on days 1, 2 or 3 after treatment initiation, respectively; and PCT as the time elapsing from treatment initiation until complete clearance of peripheral parasitaemia.

### Parasite genotyping

Finger pricked blood samples were spotted on 3MM Whatman^(R)^ filter paper on days 0–3, 7, 14, 21, 28, in 2009–2010, and additionally on days 35 and 42 in 2014–2015, and at the time of treatment failure for parasite genotyping. The filter papers were air-dried, labelled, and stored in individual envelope bags with desiccant in order to avoid moisture and fungi growth that could destroy the integrity of the collected samples until analyzed. PCR parasite genotyping pre- and post-treatment initiation was done using *P. falciparum* merozoite surface protein 1 or 2 (MSP 1 or MSP 2) or both genes as previously described [15, 16, 22]. Briefly, block 2 of the merozoite surface protein-1 (MSP-1) and the block 3 of the merozoite surface protein-2 (MSP-2) genes, were amplified by two rounds of polymerase chain reaction (PCR) using specific primers [23]. Five microliters of the nested PCR product were resolved by electrophoresis on a 2% agarose gel and sized against 100-basepair molecular weight DNA ladder (New England Biolabs, Beverly, MA).

Parasite DNA banding patterns 3 days after treatment initiation and that at the time of recurrent parasitaemia were compared side by side with the corresponding pre-treatment DNA bands to detect actual delay in asexual parasite clearance and to compare parasite populations structures in samples, as well as to differentiate recrudescence from re-infection, respectively. Banding patterns were binned in 20 bp using the GBox and the genetic analyzer software. Post-treatment and primary infection parasites showing identical banding patterns at both MSP-1 and MSP-2 loci were considered as recrudescence, whereas non-identity in banding patterns in at least one targeted locus of MSP-1 or MSP-2 was considered as newly acquired infections. In order to confirm the absence of recurrent parasitaemia, samples obtained from one in every four patients with microscopically negative blood films were also subjected to PCR analysis. An infection was considered polyclonal if it contained multiple clones (measured by amplicon fragment sizes) per allelic family on gel electrophoresis resolution of the parasite DNA. Multiplicity of infection was defined as the number of difference alleles per infection detected in positive samples of a population.

### Kinetic evaluation of time-course of parasitaemia following treatment initiation

In the later studies (Clinical Trial Registration Numbers PACTR201508001188143, 3 July 2015; PACTR201508001191898, 7 July 2015 and PACTR201508001193368, 8 July 2015) in 360 children aged 6–191 months enrolled in Ibadan site alone in parallel studies during the same time period (2009–2010 [*n* = 254] and 2012–2015 [*n* = 106]), clinical and parasitological evaluations were done at the following times: pre-treatment (0), 1, 2, 4, 6, 8 and 24 h and on days 2–7, 14, 21, 28, 35 and 42 post-treatment initiation. The kinetics of the time-course of the asexual parasitaemia was estimated using a non-compartment model as previously described [16, 22, 24, 25]. Briefly, parasite densities (concentrations) versus time until complete clearance of parasitaemia were plotted on a semilogarithmic graph. Final parasite density at the time of apparent clearance was assumed to be 0.01 asexual parasites/μl of blood, a level below microscopic detection. The apparent terminal elimination rate constant (λ) was obtained by least square regression analysis of the post peak log-linear part of the parasitaemia-time curve and apparent terminal elimination half-life of parasitaemia was obtained from ln2/λ (that is, λt = 0.693).

### Data analysis

Data were analyzed using version 6 of Epi-Info software (Centers for Disease Control and Prevention, Atlanta, GA, USA) [26] and the statistical program SPSS for Windows version 22.0 (SPSS Inc., Chicago IL, USA) [27]. Variables considered in the analysis were related to the densities of *P. falciparum* asexual forms. Proportions were compared by calculating *χ*^2^ using Yates’ correction, Fisher’s exact or Mantel Haenszel tests as appropriate. Normally distributed, continuous data were compared by Student’s t test and analysis of variance (ANOVA). Post hoc comparisons of parameters between the two treatments, where necessary, were done using Tukey Honestly Significant Difference Test (Tukey HSD). Kaplan-Meier estimator and pair-wise log-rank tests were used to determine cumulative risk of recurrent parasitaemia on day 28, after the initial clearance of parasitaemia. Univariate analyses and stepwise multiple logistic regression models were used to test the association between demographic, clinical, parasitological or haematological parameters and parasite positivity rates one or 2 days post-treatment initiation and recurrent parasitaemia following treatments and the independent predictors of these parameters, respectively. Data were double entered serially using patients’ codes and were only analysed at the end of the study. All test of significance were two-tailed and *P* values < 0.05 were taken to indicate significant differences.

## Results

### Study cohort

In the initial studies, between October 2009 and December 2015, we enrolled 1341 children for the efficacy studies (AL [*n* = 663] and AA [*n* = 678]) (Table 1). One 34 months old male treated with AA had early treatment failure and another 31 months old female treated with AL developed danger signs within 1 day of enrolment in 2009–2010. In 2014–2015, 3 children had early treatment failure (two males aged 9 months and 24 months treated with AA and one female aged 24 months treated with AL) developed danger signs within 1 day of enrolment). Table 1 shows the baseline characteristics of the enrolled children according to treatment group and period of enrolment. Overall, children enrolled at the end of the second 5 years of deployment (2014–2015) were significantly older (*P* <  0.0001), had significantly lower body temperature (*P* = 0.005), significantly higher haematocrit (*P* = 0.04) and significantly lower proportion of children with gametocyte carriage (*P* <  0.0001) compared with those enrolled at the end of the first 5 years of deployment (2009–2010) (Table 1). Other parameters such as gender, weight, proportion with anaemia at presentation, enrolment parasitaemia were similar between the two enrolment periods.

**Table 1:** Baseline characteristics of 1341 children enrolled in the efficacy study

Variables	All treatments			*P* value	Artemether-lumefantrine		*P* value	Artesunate-amodiaquine		*P* value
	2009–2010 (*n* = 743)	2014–2015 (*n* = 598)	All (*n* = 1341)		2009–2010 (*n* = 360)	2014–2015 (*n* = 303)		2009–2010 (*n* = 383)	2014–2015 (*n* = 295)	
Male∶Female	414∶329	312∶286	726∶615	0.22	201∶159	159∶144	0.43	213∶170	153∶142	0.39
Age (month)										
Mean	36.3	39.8	37.8	< 0.0001	36	40.5	0.001	36.5	39	0.04
95% *CI*	35.1–37.4	38.4–41.1	37–38.7		34.2–37.8	38.6–42.3		35–38.1	37.2–40.9	
Number ≤ 24 months (%)	237 (31.9)	141 (23.6)	378 (28.2)	0.001	123 (34.2)	68 (22.4)	0.001	114 (29.8)	73 (24.7)	0.18
Weight (kg)										
Mean	13.3	13.5	13.4	0.56	13.2	13.5	0.34	13.5	13.4	0.89
95% *CI*	13–13.6	13.1–14	13.2–13.6		12.7–13.7	13–14		13.1–13.9	13–14	
Temperature (°C)										
Mean	38	37.8	37.9	0.005	38	37.9	0.12	38	37.8	0.02
95% *CI*	37.9–38.1	37.7–37.9	37.9–38		37.9–38.1	37.8–38		37.9–38	37.6–37.9	
Number with										
≥ 37.5 °C (%)	556 (74.8)	377 (64)	933 (69.6)	< 0.0001	267 (74.2)	195 (64.4)	0.008	289 (75.5)	182 (64.4)	< 0.0001
≥ 40 °C (%)	25 (3.4)	53 (8.9)	78 (5.8)	< 0.0001	13 (3.6)	22 (7.3)	0.06	12 (3.1)	31 (7.3)	< 0.0001
Haematocrit (%)										
Mean	29.8	30.4	30.1	0.04	29.7	30.3	0.11	29.9	30.5	0.17
95% *CI*	29.4–30.2	30–30.8	29.8–30.4		29.1–30.2	30–30.8		29.4–30.5	29.9–31.1	
Number with anaemia (%)	294 (39.6)	233 (39)	527 (39.3)	0.15	142 (39.4)	120(39.6)	0.43	152 (39.7)	113 (39.6)	0.24
(i) Mild (%)	258 (34.7)	216 (36.1)	474 (35.3)	0.64	124 (34.4)	110 (36.3)	0.68	134 (35)	106 (36.3)	0.84
(ii) Moderate (%)	34 (4.6)	16 (2.7)	50 (3.7)	0.09	18 (5)	10 (3.3)	0.37	16 (4.2)	6 (3.3)	0.18
(iii) Severe (%)	2 (0.3)	1 (0.2)	3 (0.2)	1.0	0 (0)	0 (0)	–	2 (0.5)	1 (0)	1.0
Parasitaemia (μl^−1^)										
Geometric mean	14 733	15 887	15 237	0.4	15 169	16 250	0.65	14 337	15 519	0.48
95% *CI*	13 108–16 239	14 211–17 760	14 050–16 403		12 993–17 710	13 938–18		12 201–16 413	13 200–18 244	
> 100 000 μl^− 1^ (%)	71 (9.6)	60 (10)	131 (9.8)	0.83	37 (10.3)	956 30 (9.9)	0.95	34 (8.9)	30 (10.2)	0.65
Gametocytaemia (%)	159 (21.4)	29 (4.8)	188 (14)	< 0.0001	82 (22.8)	9 (3)	< 0.0001	77 (20.1)	20 (6.8)	< 0.0001

#### Transmission during period of observation

Overall, parasite rate during the study period was 35.5% (4236 of 11 930 children) and it did not differ during the two study periods (35% [1826 of 5217 children] in 2009–2010 versus 35.9% [2410 of 6713 children] in 2014–2015). Gametocyte carriage declined significantly during the two study periods (see below). Data on other indices of transmission intensity during the period of observation were not evaluated.

### Clinical responses

#### Fever clearance

Treatment with AA cleared fever significantly faster than AL [mean of 1.09 days (95% *CI*: 1.06–1.12, *n* = 470) versus mean of 1.2 days (95% *CI*: 1.15–1.25, *n* = 462) respectively, *P* <  0.0001]. Time to clear fever rose significantly (*P* = 0.002) from a mean of 1.1 days (95% *CI*: 1.08–1.14, *n* = 555) in 2009–2010 to a mean of 1.2 days (95% *CI*: 1.2–1.3, *n* = 377) in 2014–2015 (Table 2). Post hoc analysis showed an increase in fever clearance time in 2014–2015 in AL- (*P* = 0.002) but not in AA-treated children (*P* = 0.36) compared with 2009–2010 (Table 2).

**Table 2:** Therapeutic responses in malarious children following artemisinin-based combination chemotherapies

Variables	All treatments			*P* value	Artemether-lumefantrine		*P* value	Artesunate-amodiaquine		*P* value
	2009–2010 (*n* = 743)	2014–2015 (*n* = 598)	All (*n* = 1341)		2009–2010 (*n* = 360)	2014–2015 (*n* = 303)		2009–2010 (*n* = 384)	2014–2015 (*n* = 295)	
Clinical response										
FCT (day)										
Mean	1.1	1.2	1.2	0.002	1.1	1.3	0.002	1.1	1.1	0.36
95% *CI*	1.08–1.14	1.2–1.3	1.1–1.2		1.1–1.2	1.2–1.4		1.05–1.12	1.1–1.2	
Parasitological responses										
APPD1 (%)	398 (53.6)	370 (61.9)	768 (57.3)	0.002	210 (58.3)	199 (65.7)	0.06	188 (49.1)	171 (58)	0.02
APPD2 (%)	38 (5.1)	153 (25.6)	191 (14.2)	< 0.0001	21 (5.8)	83 (27.4)	< 0.0001	17 (4.4)	70 (23.7)	< 0.0001
APPD3 (%)	7 (0.9)	14 (2.3)	21 (1.6)	0.07	2 (0.6)	9 (3)	0.03	5 (1.3)	5 (1.7)	0.92
PRRD1										
Geometric mean	444	409	428	0.75	318	270	0.63	609	627	0.86
95% *CI*	349–565	315–531	359–511		222–454	186–391		440–841	438–898	
PRRD2										
Geometric mean	7594	11136	4723	< 0.0001	11112	4439	0.0002	11 158	5034	0.007
95% *CI*	6709–8597	9742–12729	3802–5868		9092–13 581	3276–6016		9335–13 337	3691–6864	
PCT (day)										
Mean	1.6	1.9	1.8	< 0.0001	1.7	2	< 0.0001	1.6	1.9	< 0.0001
95% *CI*	1.55–1.64	1.9–2.0	1.7–1.8		1.6–1.7	1.9–2.1		1.5–1.6	1.8–2.0	
Haematological response										
AnRT (day)										
Mean	10.6	10.1	10.4	0.53	11.1	11	0.9	10	9.2	0.4
95% *CI*	9.7–11.5	9.0–11.2	9.7–11.1		9.8–12.5	9.3–12.8		8.8–11.3	7.8–10.7	

*FCT* Fever clearance time, *APPD1*: Asexual parasite positivity 1 day post-treatment initiation, *APPD2* Asexual parasite positivity 2 days post-treatment initiation, *APPD3* Asexual parasite positivity 3 days post-treatment initiation, *PRRD1* Parasite reduction ratio 1 day post-treatment initiation, *PRRD2* Parasite reduction ratio 2 days post-treatment initiation, *PCT* Parasite clearance time, *AnRT* Anaemia recovery time

#### Adverse events

The proportions of children reporting adverse events in the first week of initiating treatments were similar during the two time periods (78 of 223 children [35%] versus 47 of 117 children [37%] in 2009–2010 and 2014–2015, respectively, *P* = 0.75).

### Parasitological treatment responses

#### Residual asexual parasitaemia 1 day after treatment initiation (APPD1)

In all children, there was no expansion of baseline asexual parasitaemia 1 day after treatment initiation. When data from both treatment groups were pooled together, there was a striking increase in the proportion of children with residual asexual parasitaemia 1 day after treatment initiation between 2009 and 2010 and 2014–2015 (from 53.6% [398 of 743 children] to 61.9% [370 of 598 children], *P* = 0.002). The increase was significant with AA treatment (49% [188 of 384 children] versus 58% [171 of 295 children], *P* = 0.02]) but not with AL treatment (58.3% [210 of 360 children] versus 65.7% [199 of 303 children], *P* = 0.06).

#### Multivariate analysis of residual asexual parasitaemia (APPD1)

In a multivariate logistic regression model, haematocrit > 27% 1 day after treatment initiation, enrolment parasitaemia > 75 000 μl^− 1^, treatment with AL and enrolment in 2014–2015 independently predicted residual APPD1 (a*OR* ≥ 1.6 and *P* ≤ 0.01, Table 3). When patients treated with AL were analysed separately, enrolment body temperature > 40 °C (a*OR* = 2.8, 95% *CI*: 1.1–6.9, *P* = 0.03), enrolment haematocrit > 32% (a*OR* = 1.4, 95% *CI*: 1.0–2.0, *P* = 0.048) and asexual parasitaemia > 75 000 μl^− 1^ (a*OR* = 1.7, 95% *CI*: 1.0–2.9, *P* = 0.04) independently predicted APPD1. When patients treated with AA were analysed separately, haematocrit > 27% 1 day after treatment initiation (a*OR* = 2.4, 95% *CI*: 1.6–3.7, *P* <  0.0001), enrolment parasitaemia > 75 000 μl^− 1^ (a*OR* = 1.9, 95% *CI*: 1.1–3.2, *P* = 0.02) and enrolment in 2014–2015 (a*OR* = 1.8, 95% *CI*: 1.2–2.7, *P* = 0.002) independently predicted APPD1.

**Table 3:** Predictors of residual asexual parasitaemia 1 day post-initiation of artemisinin-based combination treatments in acutely malarious children

Variable	Total number	Number with APPD1	*OR* (95% *CI*)	*P* value	a*OR* (95% *CI*)	*P* value
Gender						
Female	615	339	1			
Male	726	429	1.2 (0.9–1.5)	0.16	–	–
Age (month)						
> 24	963	550	1			
≤ 24	378	218	1.0 (0.8–1.3)	0.9	–	–
Temperature at presentation (°C)						
≤ 37.4	409	234	1			
> 37.4	932	534	1.0 (0.8–1.3)	1.0	–	–
Fever on day 1						
Absent	1189	712	1			
Present	132	82	1.2 (0.8–1.8)	0.33	–	–
Haematocrit at presentation (%)						
< 30	728	425	1			
≥ 30	526	288	0.9 (0.7–1.2)	0.22	–	–
Haematocrit on day 1 (%)						
≤ 27	275	140	1		1	
> 27	640	384	1.4 (1.1–1.9)	0.01	1.6 (1.2–2.1)	0.002
Enrolment Parasitaemia (μl^−1^)						
≤ 75 000	1148	631	1		1	
> 75 000	193	137	2.0 (1.3–3)	< 0.0001	1.9 (1.3–2.7)	0.002
Gametocytaemia						
Absent	1029	593	1			
Present	188	115	1.2 (0.8–1.6)	0.41	–	–
Drug treatment						
AA	678	359	1			
AL	663	409	1.4 (1.2–1.8)	0.001	1.8 (1.1–2.8)	0.01
Period of enrolment						
2009–2010	743	398	1			
2014–2015	598	370	1.4 (1.1–1.8)	0.003	1.8 (1.4–2.4)	< 0.0001

*APPD1* Asexual parasite positivity 1 day post-treatment initiation, *OR* Odd ratio, a*OR* Adjusted odd ratio, *CI* Confidence interval, *AL* Artemether-lumefantrine, *AA* Artesunate-amodiaquine

#### Residual asexual parasitaemia 2 days after treatment initiation (APPD2)

The proportion of children with residual asexual parasitaemia 2 days after treatment initiation rose from 5.1% (38 of 743 children) in 2009–2010 to 25.6% (153 of 598 children) in 2014–2015 (*P* <  0.0001) (Table 2). In a post hoc analysis, the proportions of children with APPD2 rose significantly from 5.8 to 27.4% and from 4.4 to 23.7% in AL- and AA-treated children, respectively, (*P* <  0.0001 for each, Table 2) during the same period.

#### Multivariate analysis of residual APPD2

In a multivariate logistic regression model, fever 1 day after treatment initiation, haematocrit > 29% 1 day after treatment initiation, APPD1, and enrolment in 2014–2015 independently predicted residual APPD2 (a*OR* > 1.6 and *P* ≤ 0.03, Table 4]. When patients treated with AL were analysed separately, asexual parasitaemia 1 day after treatment initiation (a*OR* = 8.5, 95% *CI*: 4.0–18.1, *P* <  0.0001) and enrolment in 2014–2015 (a*OR* = 3.7 (95% *CI*: 2.1–6.5), *P* <  0.0001) independently predicted APPD2. When patients treated with AA were analysed separately, haematocrit > 29% 1 day after treatment initiation (a*OR* = 2.6, 95% *CI*: 1.4–4.7, *P* = 0.002), residual asexual parasitaemia 1 day after treatment initiation (a*OR* = 10.8, 95% *CI*: 4.8–24.4, *P* <  0.0001) and enrolment in 2014–2015 (a*OR* = 5.2, 95% *CI*: 2.5–10.8, *P* <  0.0001) independently predicted APPD2.

**Table 4:** Predictors of residual asexual parasitaemia 2 days post-initiation of artemisinin-based combination treatments in acutely malarious children

Variable	Total number	Number with APPD2	*OR* (95% *CI*)	*P* value	a*OR* (95% *CI*)	*P* value
Gender						
Female	615	92	1			
Male	726	99	0.9 (0.7–1.2)	0.54	–	–
Age [month]						
> 24	963	138	1			
≤ 24	378	53	1.0 (0.7–1.4)	0.95	–	–
Temperature at presentation (°C)						
≤ 40	1263	172	1			
> 40	78	19	2.0 (1.2–3.5)	0.01	1.0 (0.5–1.9)	0.97
Fever on day 1						
Absent	1189	152	1			
Present	132	32	2.2 (1.4–3.4)	0.001	1.8 (1.1–3.2)	0.03
Haematocrit at presentation (%)						
< 30	728	115	1			
≥ 30	526	65	0.8 (0.5–1.0)	0.1	–	–
Haematocrit on day 1 (%)						
≤ 29	405	58	1		1	
> 29	510	102	1.5 (1.1–2.1)	0.01	1.7 (1.1–2.5)	0.01
Enrolment Parasitaemia (μl^−1^)						
≤ 100 000	1211	162	1		1	
> 100 000	130	29	1.9 (1.2–2.9)	0.008	1.4 (0.8–2.5)	0.22
Parasitaemia on day 1						
Absent	573	19	1		1	
Present	768	172	8.4 (5.2–13.7)	< 0.0001	9.3 (5.2–16.6)	< 0.0001
Gametocytaemia						
Present	188	13	1			
Absent	1029	178	2.8 (1.6–5.1)	< 0.0001	1.4 (0.5–3.5)	0.53
Drug treatment						
AA	678	87	1			
AL	663	104	1.3 (0.9–1.7)	0.16	–	–
Period of enrolment						
2009–2010	743	38	1			
2014–2015	598	153	6.4 (4.4–9.3)	< 0.0001	3.0 (1.7–5.1)	< 0.0001

*APPD2* Asexual parasite positivity 2 days post-treatment initiation, *OR* Odd ratio, a*OR* Adjusted odd ratio, *CI* Confidence interval, *AL* Artemether-lumefantrine, *AA* Artesunate-amodiaquine

#### Residual asexual parasitaemia 3 days after treatment initiation (APPD3)

Proportion of children with PCR-confirmed residual asexual parasitaemia 3 days after treatment initiation rose from 0.9% (seven of 743 children) in 2009–2010 to 2.3% (14 of 598 children) in 2014–2015 (Table 2). In a post hoc analysis, the proportions of children with APPD3 rose significantly from 0.6 to 3% (six folds) in AL-treated children, (*P* = 0.03, Table 2) but not in AA-treated children during the same period (five of 383 children [1.3%] versus 5 of 295 children [1.7%] in 2009–2010 and 2014–2015, respectively, *P* = 0.92).

#### Parasite reduction ratio 1 day after treatment initiation (PRRD1)

Geometric mean parasite reduction ratio 1 day after treatment initiation did not change over the two study periods (444 [95% *CI*: 349–565, *n* = 743] versus 409 [95% *CI*: 315–531, *n* = 598], respectively, *P* = 0.75, in 2009–2010 and 2014–2015, respectively).

#### Parasite reduction ratio 2 days after treatment initiation

Overall for both treatments, geometric mean parasite reduction ratio 2 days after treatment initiation (GMPRRD2) dropped by 57.2% (2.3-fold lower) between 2009 and 2010 (geometric mean 11 000, 95% *CI*: 9700–13000) and 2014–2015 (geometric mean 4700, 95% *CI*: 3800–5900, *P* <  0.0001). GMPRRD2 in the AL treatment group dropped by 60% (2.5-fold lower) (geometric mean 11 000, 95% *CI*: 9100–14 000) and (geometric mean 4400, 95% *CI*: 3300–6000, *P* = 0.0002) and in the AA treatment group by 54.5% (2.2-fold lower) (geometric mean 11 000, 95% *CI*: 9300–13 000) and (geometric mean 5000, 95% *CI*: 3700–6900, *P* = 0.007) between 2009 and 2010 and 2014–2015 (Table 2).

#### Parasite clearance

Treatment with AA cleared asexual parasitaemia significantly faster than AL (mean of 1.7 days [95% *CI*: 1.6–1.8, *n* = 678] versus mean of 1.8 days [95% *CI*: 1.8–1.9, *n* = 663], respectively, *P* = 0.006). Parasite clearance time increased significantly from a mean of 1.6 days (95% *CI*: 1.55–1.64, *n* = 743) in 2009–2010 to a mean of 1.9 days (95% *CI*: 1.9–2.0, *n* = 598) in 2014–2015 (*P* <  0.0001, Table 2). Post hoc analysis showed parasite clearance time rose significantly in children treated with AA and AL during the same time period (*P* <  0.0001 for each, Table 2).

### Probability of reappearance of asexual parasitaemia following initial clearance

**Fig. 3:**
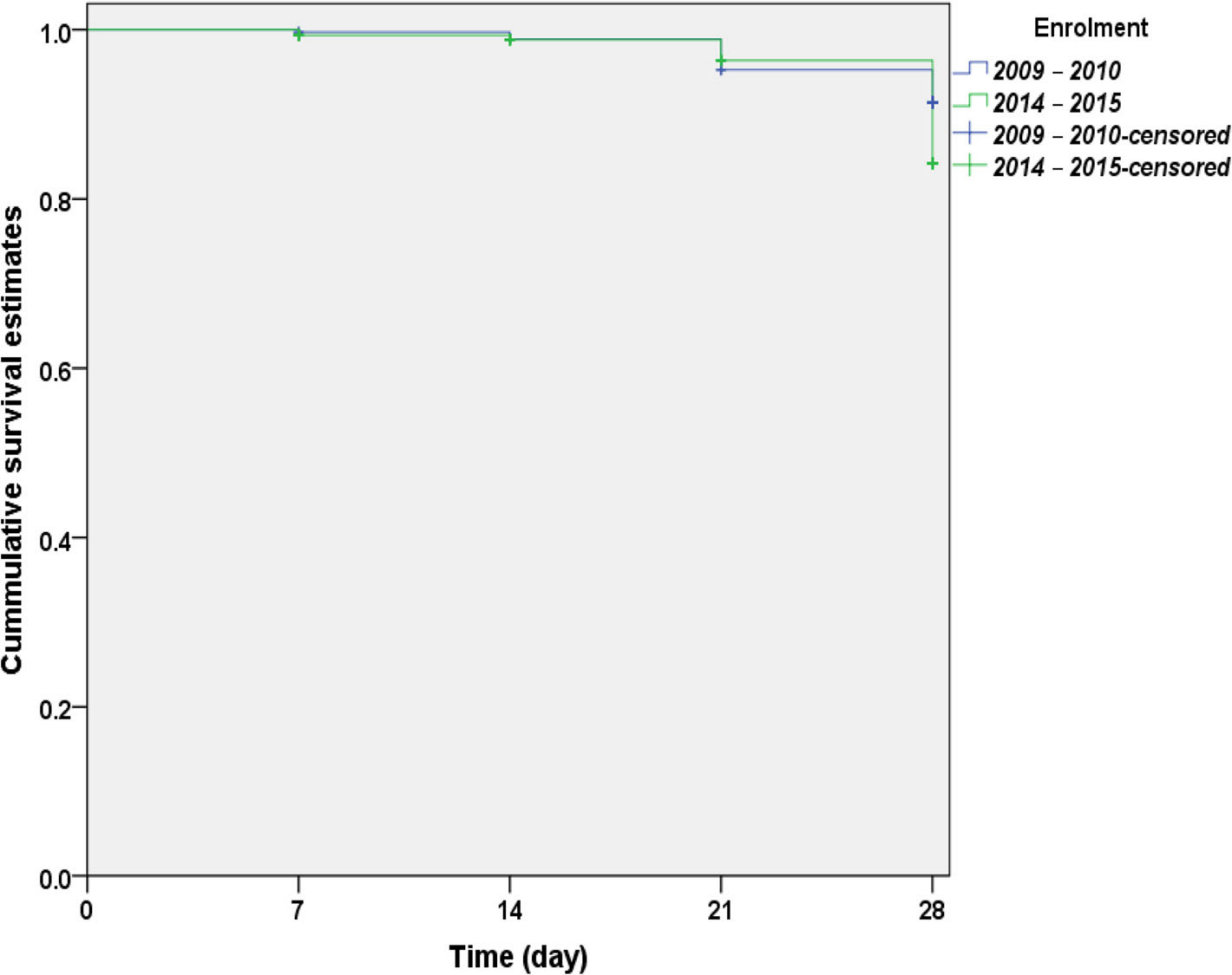
Kaplan-Meier survival estimates of reappearance of asexual parasitaemia after initial clearance following artemisinin-based combination treatments in 2009–2010 (blue line) and 2014–2015 (green line). Log-rank statistic = 14.12, *P* = 0.0002. Pooled analysis of artemether-lumefantrine and artesunate-amodiaquine treatments

By day 28, in a pooled analysis of both treatments, probability of recurrent asexual parasitaemia following initial clearance was significantly higher in children enrolled in 2014–2015 compared with 2009–2010 (Log rank statistic = 14.12, *P* = 0.0002, Fig. 3). When patients treated with AL and AA were analysed separately, the probabilities of recurrent asexual parasitaemia were significantly higher in 2014–2015 compared with 2009–2010 (Log-rank statistic = 7.8 and 5.64, *P* = 0.020 and 0.005, respectively).

Multivariate analysis of recurrent asexual parasitaemia within 28 days after treatment initiation: In a stepwise multivariate logistic regression model of pooled data from the two treatment groups, residual asexual parasitaemia 1 day after treatment initiation (APPD1) (a*OR* = 1.7, 95% *CI*: 1.2–2.5, *P* = 0.005), gametocyte carriage within the first week of presentation (a*OR* = 2.0 95% *CI*: 1.3–3.8, *P* = 0.001) and enrolment in 2014–2015 (a*OR* = 2.0, 95% *CI*: 1.4–2.9, *P* <  0.0001) independently predicted recurrent asexual parasitaemia within 28 days of treatment initiation.

### Probability of recrudescent parasitaemia following initial clearance

Recrudescent infections, confirmed by PCR, occurred in 34 of 731 children (4.7%) in 2009–2010 and in 16 of 544 children (2.9%) (*P* = 0.16) by day 28. In a pooled analysis of both treatments, probability of recrudescent parasitaemia following initial clearance was similar in children enrolled in 2009–2010 and in 2014–2015 (Log rank statistic = 2.78, *P* = 0.1). When patients treated with AL and AA were analysed separately, the probabilities of recrudescent parasitaemia were similar at the two time periods (Log-rank statistic = 1.14 and 1.71, *P* = 0.29 and 0.19, respectively).

### Multiplicity of infection pre-treatment initiation

Overall, data for multiplicity of infection were available in 113 children (50 children in 2009–2010 and 63 in 2014–2015) who had recurrent parasitaemia. Proportion of children with polyclonal infection increased significantly from 32% in 2009–2010 (16 of 50 children) to 57% in 2014–2015 (36 of 63 children) (*P* = 0.01). Multiplicity of infection (MOI) also increased significantly from 1.3 (95% *CI*: 1.2–1.5) to 2.0 (95% *CI*: 1.7–2.3) (*P* <  0.0001) during the same time period.

### Kinetic evaluation of time-course of asexual parasitaemia following treatment initiation

#### Study cohort

**Fig. 4:**
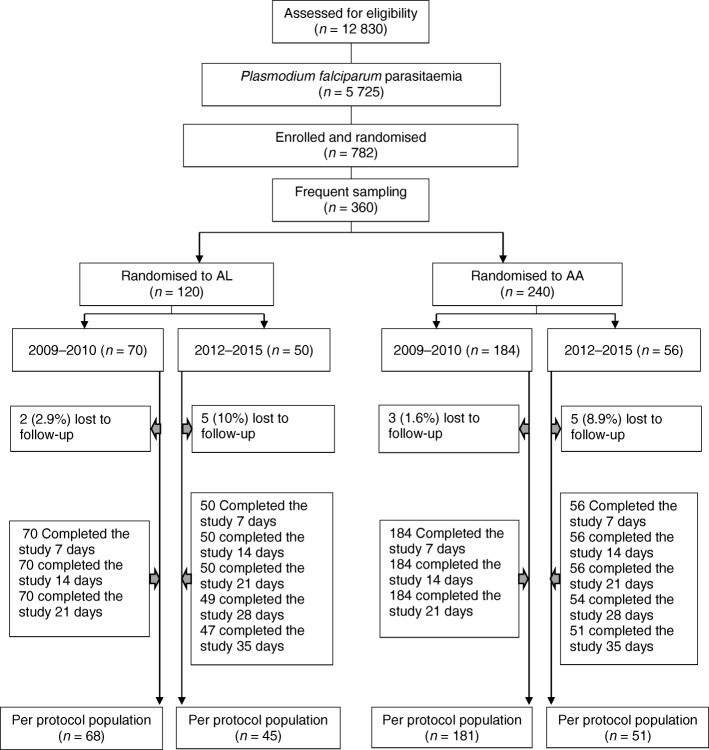
Study profile of children enrolled in parasitaemia half-life study. AA: Artesunate-amodiaquine; AL: Artemether-lumefantrine

**Fig. 5:**
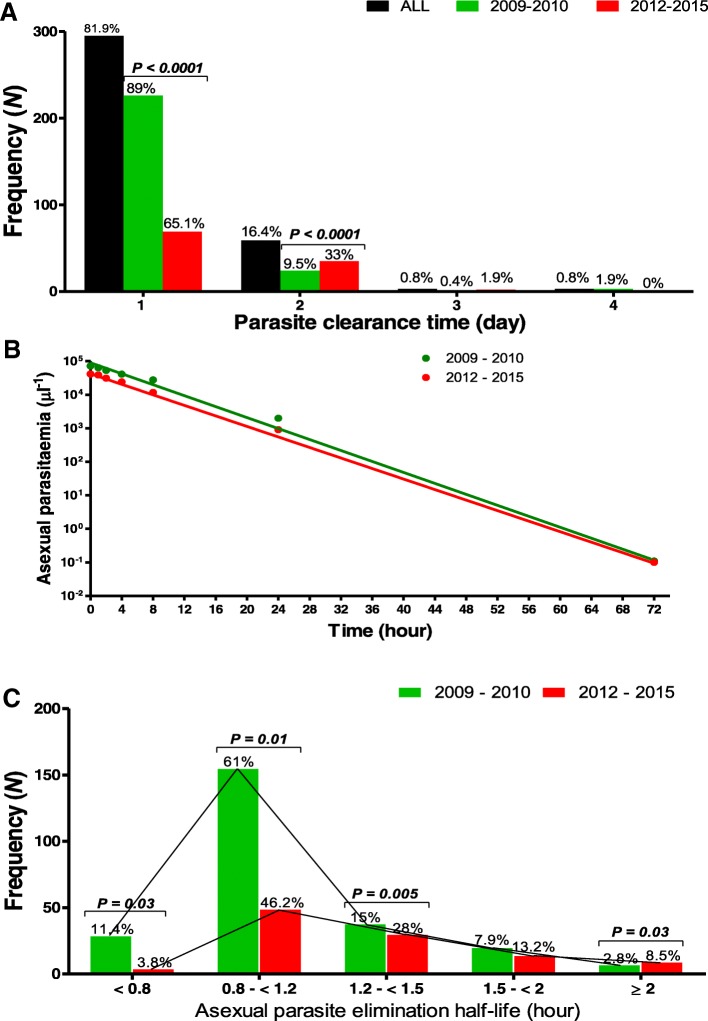
Frequency distribution of parasite clearance time (**a**) in 2009–2010 (green plots) and 2012–2015 (red plots), semilogarithmic plots of asexual parasitaemia versus time following treatment with artemether-lumefantrine or artesunate-amodiaquine (**b**), and frequency distribution of parasitaemia elimination half-life (**c**) in 2009–2010 (green plots) and in 2012–2015 (red plots)

Between May 2009 and July 2015 at Ibadan study site, we enrolled and randomized additional 360 children for kinetic evaluation of the time-course of parasitaemia at a ratio of 1:2 for AL and AA (AL [*n* = 120] and AA [*n* = 240]) (Fig. 4). All children had follow-up record till day 7 and were included in the analysis. At presentation, children enrolled in 2012–2015 had significantly lower body temperature (*P* = 0.009), and geometric mean asexual parasitaemia (*P* <  0.0001) compared with those enrolled in 2009–2010 (Table 5). Parasite clearance time and residual asexual parasitaemia 1 day after treatment initiation were significantly higher and PRRD1 and PRRD2 significantly lower in children enrolled in 2012–2015 compared with those enrolled in 2009–2010 (*P* <  0.0001 for each) (Table 5). Proportion of children with asexual parasite clearance time of 1 day declined significantly from 89% (226 of 254 children) in 2009–2010 to 65.1% (69 of 106 children) in 2012–2015 (*P* <  0.0001**)** and proportions of children with asexual parasite clearance time of 2 days increased significantly from 9.5% (24 of 254 children) to 33% (35 of 106 children) (*P* <  0.0001) during the same time period (Fig. 5a). The frequency distribution of parasite clearance time during the two periods was unimodal.

**Table 5:** Baseline characteristics and treatment responses of 360 children enrolled in the parasitaemia elimination half-life study

Variables	2009–2010 (*n* = 254)	2012–2015 (*n* = 106)	All (*n* = 360)	*P* value
Baseline characteristics				
Male∶Female	141∶113	53∶53	194∶166	0.4
Age (month)				
Mean	7.3	7.5	7.4	0.64
95% *CI*	6.9–7.7	6.8–8.1	7.0–7.7	
Number 5 years (%)	54 (21.3)	22 (20.8)	76 (21.1)	1.0
Temperature (°C)				
Mean	38.4	38	38.3	0.009
95% *CI*	38.2–38.5	37.8–38.3	38.2–38.4	
No. with				
≥ 37.5 °C (%)*	196 (77.2)	67 (23.6)	263 (73.1)	0.01
≥ 40 °C (%)	26 (10.2)	4 (3.8)	30 (8.3)	0.06
Haematocrit (%)				
Mean	32.1	32.1	32.1	0.98
95% *CI*	31.6–32.6	32.1–33.0	31.7–32.6	
No with anaemia (%)	60 (23.6)	25 (23.6)	85 (23.6)	1.0
Parasitaemia (μl^−1^)				
Geometric mean	71 393	41 404	60 811	< 0.0001
Range	1800–1 096 636	2220–454 875	1800–1 096 636	
> 100 000 μl^− 1^ (%)	165 (65)	83 (78.3)	248 (68.9)	0.02
Gametocytaemia (%)	7 (2.8)	2 (1.9)	9 (2.5)	1.0
Treatment responses				
Fever clearance time (day)				
Mean (95% *CI*)	1.1 (1.0–1.1)	1.1 (1.0–1.2)	1.1 (1.05–1.13)	0.26
Parasite clearance time (day)				
Mean (95% *CI*)	1.1 (1.1–1.2)	1.4 (1.3–1.5)	1.2 (1.2–1.3)	< 0.0001
APPD1 (%)	28 (11)	37 (34.9)	65 (18.1)	< 0.0001
APPD2 (%)	4 (1.6)	2 (1.9)	6 (1.7)	1.0
APPD3 (%)	3 (1.2)	0 (0)	3 (0.8)	-
Parasite reduction ratio 1 day				
Geometric mean	3.3 × 10^4^	3.9 × 10^3^	1.7 × 10^4^	< 0.0001
Range	2.7 × 10^0^–1.1 × 10^6^	8.0 × 10^−1^–2.4 × 10^5^	8.0 × 10^−1^–1.1 × 10^6^	
Parasite reduction ratio 2 days				
Geometric mean	6.6 × 10^4^	3.8 × 10^4^	5.6 × 10^4^	< 0.0001
Range	1.6 × 10^1^–1.1 × 10^6^	1.8 × 10^3^–3.6 × 10^5^	1.6 × 10^1^–1.1 × 10^6^	

*APPD1* Asexual parasite positivity 1 day post-treatment initiation, *APPD2* Asexual parasite positivity 2 days post-treatment initiation, *APPD3* Asexual parasite positivity 3 days post-treatment initiation, *CI* Confidential interval

### Terminal elimination half-time of asexual parasitaemia

Estimated terminal elimination half-time of asexual parasitaemia increased significantly from a mean of 1.1 h (95% *CI*: 1.1–1.2, *n* = 254) in 2009–2010 to 1.3 h (95% *CI*: 1.2–1.4, *n* = 106) in 2012–2015 (*P* <  0.0001) (Fig. 5b). When analysed according to age groups, in 6–59 month-olds, terminal elimination half-time of asexual parasitaemia increased significantly from a mean of 1.1 h (95% *CI*: 1.1–1.2, *n* = 73) in 2009–2010 to 1.4 h (95% *CI*: 1.3–1.6, *n* = 27) in 2012–2015 (*P* = 0.004). In children older than 59 months, terminal elimination half time of asexual parasitaemia also increased significantly from a mean of 1.1 h (95% *CI*: 1.1–1.2, *n* = 181) in 2009–2010 to 1.3 h (95% *CI*: 1.2–1.4, *n* = 79) in 2012–2015 (*P* = 0.001). There was no difference in mean terminal elimination half-times of asexual parasitaemia in the < 5 and > 5 year-olds (*P* = 0.68). Mean estimated terminal elimination half-times of asexual parasitaemia was similar in the two treatment groups (1.2 h [95% *CI*: 1.1–1.2, *n* = 120] versus 1.2 h [95% *CI*: 1.1–1.2, *n* = 240] in AL and AA, respectively, *P* = 0.94). There was a significantly positive correlation between parasite clearance time and terminal elimination half-time of asexual parasitaemia (r = 0.64, *P* <  0.0001, *n* = 360).

### Frequency distribution of terminal elimination half-times of asexual parasitaemia and parasite clearance times

The frequency distribution of terminal elimination half times of asexual parasitaemia was unimodal during the two study periods (Fig. 5c) as was the frequency distribution of parasite clearance times (Fig. 5a). In a pooled analyses, proportion of children with terminal elimination half-times of asexual parasitaemia ≥2 h increased significantly from 2.8% (7 of 254 children) in 2009–2010 to 8.5% (9 of 106 children) in 2012–2015 (*P* = 0.03, Fig. 5c) and proportions of children with terminal elimination half-times of asexual parasitaemia < 1.2 h decreased significantly (*P* <  0.0001) from 72.4% (184 of 254 children) to 50% (53 of 106 children) during the same time period (Fig. 5c). One 60 months old male had a terminal elimination half-time of asexual parasitaemia of 3 h.

In a univariate analyses to determine the factors associated with an terminal elimination half-time of asexual parasitaemia > 1.5 h, namely gender (male or female), age < 5 or > 5 years, body temperature < 37.4 °C or > 37.4 °C, presence or absence of fever 1 day after treatment initiation, haematocrit < 30% or ≥ 30%, enrolment parasitaemia ≤100 000 μl^− 1^ or > 100 000 μl^− 1^, treatment with AL or AA, enrolment in 2009–2010 or 2012–2015, only enrolment in 2012–2015 was significantly associated with a terminal elimination half-time of asexual parasitaemia ≥1.5 h **(**27 of 254 [11%] versus 23 of 106 [22%], *OR* = 2.3, 95% *CI*: 1.3–4.3, *P* = 0.009].

## Discussion

In these country-wide studies of the efficacies of artemisinin-based combination therapies during a 10-year period of deployment as first-line antimalarials in Nigeria, we report a significant decline of early responses of childhood *P. falciparum* infections to AL and AA. Parasite positivity 1 day after treatment initiation increased insignificantly from 58 to 66% over time in children receiving AL and significantly from 50 to 58% over time in those receiving AA. In addition, parasite positivity 2 days after initiating both treatments increased significantly from 5 to 26% over time. These increases indicate declining or worsening of early response markers, which are thought to be primarily dependent on the artemisinin components of ACTs [28, 29]. Although the magnitude of the decline in early response markers is much smaller than in the GMS [1–7, 30], the substantially increased APPD3 over time in those treated with AL is reminiscent of the situation in the GMS where declining responsiveness was a prelude to development of artemisinin-resistance in *P. falciparum* [3, 30]. For both ACTs, the substantial increase in APPD3 from 0.9 to 2.3% (2.6 folds) over time is striking and calls for a formal evaluation, in vivo, of artemisinin efficacy test of delayed parasite clearance that is devoid of confounders by artemisinin derivatives partner drugs in children under 5-year-old from these endemic areas as was carried out in some malaria transmission settings [9, 30].

Increment in APPD1 over time is common to all areas with declining responsiveness in *P. falciparum* to ACTs [2, 30]. However, it is unclear why we did not observe a significant increase in APPD1 in children treated with AL. One possible reason for this observation is that the relatively long period between deployment in 2005 and our first study in 2009–2010 masked the incremental change which probably occurred earlier than 2009–2010. To support this explanation, are the findings both APPD1 and terminal elimination half-time of asexual parasitaemia increased significantly with both ACTs when interval between the study periods was only 2 years (Table 5 and Fig. 5b).

There was remarkable similarity between some of the independent predictors of APPD1 and APPD2 such as haematocrit ≥27% and enrolment in 2014–2015 but there was no expansion of baseline asexual parasitaemia, stable baseline asexual parasitaemia, or less than 75% decrease in baseline asexual parasitaemia that would have indicated marked reduction of response to the two ACTs in any of the patients 1 day after treatment initiation. It is surprising that in contradistinction to the situation in some countries in the GMS, where declining responsiveness is associated with increased gametocyte carriage and transmission of parasites with reduced susceptibilities to ACTs [3], gametocyte carriage decreased significantly during the period of deployment in the cohorts of children we evaluated. This is explicable in the context of small but significant decline in responsiveness overtime that has not reached the critical stage for the emergence of resistance and its associated increasing gametocyte carriage. However, gametocytaemia within 1 week of initiating treatment and enrolment in 2014–2015 independently predicted recurrent asexual parasitaemia within 28 days of treatment initiation. These findings would suggest declining gametocyte carriage over time alone would be insufficient to prevent transmission of gametocytes arising from parasites with reduced susceptibility should resistance develop in the future to any of the two ACTs evaluated. It would also appear from the results of the multiple logistic regression models, enrolment in 2014–2015 may be synonymous with declining responsiveness to the two ACTs. This is not surprising as 2014–2015 may be a watershed to progression or otherwise of reduced susceptibility.

Parasites with alleles that confer slow clearance of parasitaemia in the GMS notably *Pf*K13 C580Y, R539T, Y493H are not frequently encountered in Africa [10, 31]. However, the observation of significantly increased parasite clearance time over time raises concerns about possible emergence with time of parasites with slow clearance phenotypes similar or dissimilar to those in the GMS. Thus, it is possible, with time, parasites with slow clearance phenotypes with alleles different from those in the GMS may emerge from indigenous *P. falciparum* populations in Africa as has been recently described in a non-immune visitor to Equatorial Guinea [32].

Four points may help explain the possibility of emergence with time of parasites with slow clearance phenotypes in endemic areas of Nigeria: First, an increase in APPD3 from a baseline of 0.9 to 2.3% over time is unlikely to be a random phenomenon. Second, the significantly increased risk of recurrent parasitaemia over-time, in the presence of the long acting partner drugs, may contribute to parasite survival in the presence of less than optimal parasiticidal drug concentrations [33, 34], even when PCR-corrected 28 days efficacy rates are high. Third, in many endemic areas as opposed to areas of low transmission, parasite burdens in less than 5-year olds and the probabilities of low dosing which may permit de novo selection of resistant parasites are high [33, 34]. Finally, a 20% increase in terminal elimination half-time of asexual parasitaemia in 2 years in these endemic areas of full ACTs sensitivity [15, 16, 21], when combined with significantly increased risk of recurrent parasitaemia may signal downward spiral of the declining responsiveness within a relatively short time frame.

Estimation of parasite clearance half-life is thought to be the best in vivo measure of artemisinin drug effect [29]. Although the significant increments in terminal elimination half-times of asexual parasitaemia over time in the cohorts of both young and older children we evaluated were not as marked as those reported from GMS [5, 6, 30], the data provided compelling evidence of declining parasite clearance rate manifested as significant increases in both the frequency of terminal elimination half-times of asexual parasitaemia ≥2 h from 2.8 to 8.5%, and significant reduction of frequency of terminal elimination half times of asexual parasitaemia of ≤0.8 h from 11 to 4% within a short period of 2 years (Fig. 5c). In many GMS countries, where resistance to artemisinin has developed, for example in southern Myanmar [5, 30], frequency distribution of parasite clearance time and terminal elimination half-life of parasitaemia is bimodal. The unimodal frequency distribution of terminal elimination half-times of asexual parasitaemia in the cohort of children we evaluated supports an earlier report of apparent absence of parasites with slow clearance phenotype in endemic areas of Nigeria [10]. However, it is plausible to expect that with increasing ‘right shift’ in frequency distribution of terminal elimination half-times of asexual parasitaemia over time, it is likely a bimodal distribution will emerge in the future with slow clearance parasite phenotypes.

In order not to overestimate terminal elimination half-times of asexual parasitaemia or misinterpret the observed unimodal frequency distribution of terminal elimination half-times of asexual parasitaemia, frequent samples were obtained from the patients. The limitations of our study in this context are the non-estimation of concentrations of the artemisinin components of the administered ACTs and/or their metabolites, and apart from age, the non-evaluation of the many host factors that may influence parasite clearance times such as immune status. ACTs may mobilize asexual parasites from the deep tissue into peripheral blood in the early hours following initiation of treatments in drug sensitive infections in children from endemic areas of Africa [9, 22]. This action may increase parasite clearance time when compared with those without mobilization [22]. It is unclear how this phenomenon would influence terminal elimination half-times of asexual parasitaemia in those with this phenomenon; we did not evaluate this phenomenon in our post hoc analyses.

Individual analysis of the two studies (2009–2010 and 2014–2015) showed spatial heterogeneity in clearance indices. For example, for both studies, recrudescence and residual parasitaemias on days 1 and 2 were significantly higher on the eastern compared with western flank of the study sites [15, 16]. In addition, if resistance were to develop in the future, it would more like emerge from the eastern compared to the western flank of the study sites and to occur in AL- compared to AA-treated children. However, our post hoc analyses of the two studies were not sufficiently powered to detect spatial heterogeneity of the clearance indices.

Although we did not evaluate immunity in the cohorts of children in the post hoc analyses, the limited data on transmission intensity suggested this did not change over time. The significantly increased rate of polyclonal infections and multiplicity of infection in the absence of presumed decrease in immunity, may support, in part, the emergence of a population of parasites with reduced susceptibility to the two ACTs. In a similar study of two ACTs in similarly aged Kenyan children, in an area of seasonal but intense transmission, Borrmann and others [12] found that significant decline in responsiveness over a relatively short period of time was accompanied by decrease in immunity of the children.

Overall, the critical questions are: are the present observations of post hoc analyses, an unmasking of innate reduced in vitro susceptibility of *P. falciparum* isolates in Nigeria to artemisinin first reported in the 1992, 13 years before the adoption and deployment of artemisinin-based combination therapies [35]? Is a relatively recent report of parasite isolates with reduced in vitro susceptibility to artemether from the same endemic area and their association with transporter genes [36] an indirect confirmation of the 1992 observation? Taken together, the earlier observations support the present observations of post hoc analyses, and all indicate the emergence of clonal predominance of parasites with reduced susceptibility to artemisinin components of the two ACTs evaluated.

## Conclusions

In conclusion, the declining parasitological responses through time to artemether-lumefantrine and artesunate-amodiaquine raises concerns and may be due to predominance of parasites with reduced susceptibility or decreasing herd immunity in young children in endemic areas of Nigeria.

## Additional file

Additional file 1: Multilingual abstracts in the five official working languages of the United Nations. (PDF 259 kb)

## Data Availability

The dataset supporting the findings of this article is available from the corresponding author upon request.

## Abbreviations

AA: Artesunate-amodiaquine
ACT: Artemisinin-based combination therapy
AL: Artemether-lumefantrine
AnRT: Anaemia recovery time
AOR: Adjusted odd ratio
APPD1: Asexual parasite positivity one day post-treatment initiation
APPD2: Asexual parasite positivity two days post-treatment initiation
APPD3: Asexual parasite positivity three days post-treatment initiation
*CI*: Confidence interval
DNA: Deoxyribonucleic acid
GMS: Greater Mekong Subregion
MOI: Multiplicity of Infection
MSP-1: Merozoite surface protein-1
MSP-2: Merozoite surface protein-2
*OR*: Odd ratio
PCR: Polymerase chain reaction
PCT: Parasite clearance time
PRR: Parasite reduction ratio
PRRD1: Parasite reduction ratio one day post-treatment initiation
PRRD2: Parasite reduction ratio two days post-treatment initiation
