# Supplementary material for: Declining responsiveness of childhood Plasmodium falciparum infections to artemisinin-based combination treatments ten years following deployment as first-line antimalarials in Nigeria
Source: Infect Dis Poverty. 2019 Aug 6;8:69. doi: 10.1186/s40249-019-0577-x (PMC6683392; doi:10.1186/s40249-019-0577-x)
Supplement: Supplementary file 1 — Multilingual abstracts in the five official working languages of the United Nations. (PDF 259 kb) [file 40249_2019_577_MOESM1_ESM.pdf]

Translation of the abstract into the five official working languages of the United Nations

انخفاض استجابة الالتهابات الناجمة عن البلازمود المنجلي لدى الأطفال للمركبات العلاجية القائمة على مادة الأرتيميسينين بعد عشر سنوات من توزيعها في نيجيريا كمضاد أوّلي للمالاريا.

أكينتوند سوونمي، وغودوين نتادوم، وكازييم أكانو، وفولاساد إيبيرونك، وأديجيموك أبيدي، وشيمير أغومو، وأونيكيب فولارين، وغريس غبوتوشو، وكريستيان هابي، وستيفان أوغيش، وهينرييتا أوكافور، ومارتين ميريميكفو، وفيليب أغومو، وويليام أوغالا، وإسماييلا واتيلا، وأوليغبينغا موكيولو، وفينومو فينومو، وجوي إيبينبي، ونما جيا، وخوسي أمبي، وروبينسون واماندا، وجورج إيميتشيبي، و ويلينغتون أويبو، وفرانسيس أوسيه وتيميتوبي أديرويجي، وتيتيلوبي دوكونمو، وأوموبولاجي أليبيوسو، وسيريكو أمو، وأولوابونمي باسورين، وأولوبينمي ويوي، وشوكوويبوكا أوكافور، وأودافي أكبوبوري، وبايو فاتونمبي، وإيلسي أديووي، ونينا إيزيغوي وأيواد أوديوولا

الملخص

الخلفية: لقد خلق تطور وانتشار الملاريا المقاوِمة للأرتيميسينينالناجمة عن البلازمود المنجليفي منطقة الميكونغ الكبرى دون الإقليمية دافعًا إلى الاستمرار في المراقبة العالمية لفعالية العلاجات المركبة القائمة على الأرتيميسينين (ACTs). يهدف هذا التحليل إلى تقييم التغيّرات في مؤشرات الاستجابة للعلاج المبكر بعد عشر سنوات من تبنّي العلاجات المركبة القائمة على الأرتيميسينين كعلاج أولي للملاريا المنجلية غير المعقدة في نيجيريا.

الطرق: في 14 موقع رصد عبر ست مناطق جغرافية في نيجيريا، قمنا بتقييم الاستجابات للعلاج لدى 1341 طفل تحت سن الخامسة إضافةً إلى 360 طفلًا تحت سن 16 مصابين بالملاريا غير المعقدة تم إخضاعهم إلى تجارب عشوائية للأرتيميثر-لوميفانترينفي مقابلالأرتيسونات-أمودياكين على فواصل زمنية مدتها خمس سنوات من 2009-2010 إلى 2014-2015وأخرى مدتها سنتان من 2009-2010 إلى 2012-2015 على التوالي بعد التوزيع عام 2005.

النتائج: ارتفعت إيجابية الطفليات غير الجنسية (AAPD1) بعد يوم من بدء العلاج من 54% إلى 62% ومن 5% إلى 26% بعد يومين من بدء العلاج ما بين 2009-2010 و2014-2015 (P= 0.002 و P 0.0001 على التوالي). ارتفع زمن التخلص من الطفليات على نحو معتبر من 1.6 يوم (95% مجال الثقة CI [: 1.55–1.64]) إلى 1.9 يوم (95% CI: 1.9–2.0) وانخفض المتوسط الهندسي لنسبة تقليل الطفليات بعد يومين من بدء العلاج على نحو معتبر من 11000 إلى 4700 في غضون نفس الفترة الزمنية (P< 0.0001 لكل واحد). وجود الطفيليات في الدم عند الإخضاع للتحاليل > μl^{-1} 75000 ، الهيماتوكريت> 27% بعد يوم من بدء العلاج، وقد توقع العلاج بالأرتيميثر- لوميفانترين والخضوع للتجربة خلال 2014-2015 ارتفاع إيجابية الطفليات غير الجنسية على نحو مستقل. وعلى النظير من ذلك، قدّر كابلان-ماير ارتفاع خطر الالتهابات المتكرّرة عند اليوم 28 من 8% إلى 14% (P= 0.005) ومن 9% إلى 15% (P = 0.02) باستخدام الأرتيميثر-لوميفانترين والأرتيسونات- أموديكين على التوالي. ارتفع متوسط نصف العمر لوجود الطفيليات غير الجنسية من 1.1 ساعة إلى 1.3 ساعة في غضون عامين (P < 0.0001).

الاستنتاج: تشير هذه المعطيات إلى أن انخفاض استجابة الطفيليات للعلاجات المركبة القائمة على الأرتيميسينين عبر الزمن قد يكون راجعًا إلى ظهور طفيليات أقل قابلية للتأثر او انخفاض مناعة هؤلاء الأطفال ضد الالتهابات.

Translated from English version into Arabic by Walid Ghazi, Revised by Ahmed Ibrahim, through

TRANSLATORS
WITHOUT BORDERS

儿童恶性疟原虫感染对以青蒿素为基础的联合治疗的反应性下降：尼日利亚一线抗疟药物研究

Akintunde Sowunmi, Godwin Ntadom, Kazeem Akano, Folasade O. Ibironke, Adejumoke I. Ayede, Chimere Agomo, Onikepe A. Folarin, Grace O. Gbotosho, Christian Happi, Stephen Oguche, Henrietta U. Okafor, Martin Meremikwu, Philip Agomo, William Ogala, Ismaila Watila, Olugbenga Mokuolu, Finomo Finomo, Joy C. Ebenebe, Nma Jiya, Jose Ambe, Robinson Wammanda, George Emechebe, Wellington Oyibo, Francis Useh, Temitope Aderoyeje, Titilope M. Dokunmu, Omobolaji T. Alebiosu, Sikiru Amoo, Oluwabunmi K. Basorun, Olubunmi A. Wewe, Chukwuebuka Okafor, Odafe Akpoborie, Bayo Fatunmbi, Elsie O. Adewoye, Nnenna M. Ezeigwe and Ayoade Oduola

摘要

引言：大湄公河次区域恶性疟原虫的抗药性的产生和传播为继续在全球范围内监测以青蒿素为基础的联合疗法的疗效提供了动力。本研究目的是在尼日利亚采用 ACTs 作为无并发症恶性疟的一线治疗十年后，评估早期治疗反应标志物的变化。

方法：在尼日利亚六个地区的 14 个哨点，我们评估了 1341 名五岁以下儿童，以及另外 360 名未满一岁儿童的治疗反应，在 2005 年部署后，他们在 2009-2010 年和 2014-2015 年以及 2009 年至 2010 年和 2012 年至 2015 年间，分别以 5 年一次和 2 年一次参加蒿甲醚-苯芴醇与青蒿琥酯-阿莫地喹的随机试验。

结果：从 2009-2010 至 2014-2015 年，治疗开始后 1 天（APPD1）的无性寄生虫阳性率从治疗开始后的 54%上升至 62%，而治疗开始后 2 天，从 5%上升至 26%（分别为 P＝0.002 和 P <0.0001）。寄生虫清除时间从 1.6 天（95%置信区间[CI]：1.55-1.64）增加至 1.9 天（95% CI：1.9-2.0），治疗开始后 2 天几何平均寄生虫减少率从 11 000 下降至在同一时间段内 4700（每个 P <0.0001）。入组治疗后寄生虫血症> 75000μl^{-1}，血细胞比容> 27%，蒿甲醚-苯芴醇治疗和 2014 -2015 年入组是 APPD1 的独立预测因素。与此同时，Kaplan-Meier 估计第 28 天复发感染的风险均升高，分别为蒿甲醚-青蒿琥酯疗法从 8%上升到 14%（P ＝ 0.005）及青蒿琥酯-阿莫地喹的疗法从 9%上升到 15%（P＝0.02）。平均无性寄生虫血症半衰期在两年内从 1.1 小时显著增加至 1.3 小时（P <0.0001）。

结论：这些数据表明，对于两种 ACT 的寄生虫反应下降可能是由于易感性降低寄生虫的出现以及儿童感染免疫力下降所导致的。

Translated from English version into Chinese by Xin-Yu Feng

CHINESE CENTER FOR DISEASE CONTROL AND PREVENTION
NATIONAL INSTITUTE OF PARASITIC DISEASES

Diminution de la réactivité des infections infantiles à Plasmodium falciparum aux combinaisons thérapeutiques à base d'artémisinine dix ans après le déploiement en tant qu'antipaludique de première intention au Nigeria

Ntadom Sowunmi Akintunde, Godwin, Kazeem Akano, O. Folasade Ibironke, Adejumoke I., Ayede Onikepe Chimere Agomo, A. Folarin, Grace O. Gbotosho, Christian Happi, Stephen Oguche, Henrietta U. Okafor, Martin Meremikwu, Philip Agomo, William Ogala, Ismaila Watila Mokuolu Finomo, Olugbenga, Ebenebe Finomo, Joy C., nma Jiya, Jose Ambe, Wammanda Emechebe Robinson, George, Wellington Oyibo, Francis Useh, Temitope a Aderoyeje Dokunmu Titilope, M., T., Sikiru Omobolaji Alebiosu Oluwabunmi Basorun Amoo, K., A., Olubunmi Wewe Chukwuebuka Okafor, Odafe Akpoborie, Bayo Fatunmbi, Elsie O. Adewoye, Nnenna M. Ezeigwe et Oduola Ayoade.

Contexte

Résumé : Le développement et la propagation du paludisme à Plasmodium falciparum résistant à l'artémisinine dans la sous-région du Grand Mékong ont créé un élan pour la surveillance mondiale continue de l'efficacité des combinaisons thérapeutiques à base d'artémisinine (ACT). Ce post-analyses vise à évaluer les changements dans les marqueurs de la réponse au traitement précoce dix ans après l'adoption des ACT comme traitement de première intention du paludisme non compliqué à falciparum au Nigéria.

Méthodes : Dans les 14 sites sentinelles de six zones géographiques du Nigéria, nous avons évalué les réponses au traitement chez 1341 enfants de moins de cinq ans et chez 360 autres enfants de moins de seize ans atteints de paludisme sans complication inscrits à des essais randomisés d'artéméther-luméfantrine versus d'artésunate-amodiaquine à 5 ans d'intervalle en 2009-2010 et 2014-2015 et à 2 ans d'intervalle en 2009-2010 et 2012-2015, respectivement, après leur déploiement en 2005.

Résultats : La positivité du parasite asexué un jour après le début du traitement (APPD1) est passée de 5% à 26% après le début du traitement, de 54% à 62%, et de 2% après le début du traitement (P = 0,002 et P <0,0001, respectivement). Le temps d'élimination des parasites a augmenté de manière significative à partir de 1,6 jour (intervalle de confiance à 95% [IC]) : 1,55-1,64) à 1,9 jour (95 % *CI* : 1,9-2,0) et le ratio de réduction parasite moyen géométrique 2 jours après le début du traitement a considérablement diminué, passant de 11 000 à 4 700 au cours de la même période (p <0,0001 pour chacun). Parasité du recrutement> 75 000 µl-1, hématocrite> 27% un jour après le début du traitement, un traitement à l'artéméther-luméfantrine et un recrutement en 2014-2015 ont prédit de manière indépendante l'APPD1. En parallèle, Kaplan-Meier a estimé que le risque d'infections récurrentes au 28e jour était passé de 8% à 14% (p = 0,005) et de 9% à 15% (p = 0,02) avec respectivement l'artéméther-luméfantrine et l'artésunate-amodiaquin. La demi-vie moyenne de la parasitémie asexuée a considérablement augmenté, passant de 1,1 heure à 1,3 heure en l'espace de deux ans (P <0,0001).

Conclusions : Ces données indiquent une diminution des réponses parasitologiques à travers le temps pour les deux ACT, peut-être dues à l'émergence de parasites ayant une sensibilité réduite ou à la diminution de l'immunité à l'infection chez ces enfants.

Translated from English version into French by Emilie Rigault Fourcadier, Revised by Isabelle Beaumont, through

TRANSLATORS
WITHOUT BORDERS

Отсутствие восприимчивости малярийной *Plasmodium falciparum* у детей к комбинированной терапии, содержащей артемизин, спустя 10 лет после применения антималярийных средств первой линии в Нигерии

Akintunde Sowunmi, Godwin Ntadom, Kazeem Akano, Folasade O. Ibironke, Adejumoke I. Ayede, Chimere Agomo, Onikepe A. Folarin, Grace O. Gbotosho, Christian Happi, Stephen Oguche, Henrietta U. Okafor, Martin Meremikwu, Philip Agomo, William Ogala, Ismaila Watila, Olugbenga Mokuolu, Finomo Finomo, Joy C. Ebenebe, Nma Jiya, Jose Ambe, Robinson Wammanda, George Emechebe, Wellington Oyibo, Francis Useh, Temitope Aderoyeje, Titilope M. Dokunmu, Omobolaji T. Alebiosu, Sikiru Amoo, Oluwabunmi K. Basorun, Olubunmi A. Wewe, Chukwuebuka Okafor, Odafe Akpoborie, Bayo Fatunmbi, Elsie O. Adewoye, Nnenna M. Ezeigwe and Ayoade Oduola

Аннотация

Справочная информация: Развитие и распространение устойчивой к артемизину малярийной инфекции *Plasmodium falciparum* в субрегионе Большого Меконга послужило толчком к проведению всемирного мониторинга эффективности артемизин-комбинированной терапии (АКТ). Целью данного последующего анализа является оценка изменений в ранних маркерах ответа на лечение спустя десять лет после принятия АКТ в качестве первой линии терапии при лечении неосложненной тропической малярии в Нигерии.

Методы: В 14 консультационных центрах в шести географических регионах Нигерии мы провели оценку ответов на лечение у 1341 ребенка в возрасте до пяти лет и дополнительно у 360 детей в возрасте до 16 лет с неосложненной малярией, которые участвовали в рандомизированных испытаниях по лечению комбинацией препаратов артеметер и люмефантрин *или* артесунат и амодиахин в период сроком 5 лет в 2009–2010 гг. и 2014–2015 и в период сроком 2 года в 2009–2010 гг. и 2012–2015 гг., соответственно после получения терапии в 2005 г.

Результаты: Наличие бесполой формы паразита спустя 1 день после начала терапии (БППД1) возросло с 54 % до 62 %, а спустя 2 дня после начала терапии — с 5 % до 26 % в период с 2009–2010 гг. до 2014–2015 гг. (P = 0,002 и P < 0,0001 соответственно). Время клиренса паразита значительно увеличилось с 1,6 дней (95 % доверительного интервала [*ДИ*]: 1,55–1,64) до 1,9 дней (95 % *ДИ*: 1,9–2,0) и соотношение средней геометрической при снижении активности паразита спустя 2 дня после начала терапии значительно снизилось с 11 000 до 4700 в течение одинакового периода времени (P <0,0001 для каждого). Увеличение уровня паразитемии > 75 000 μl^{-1}, гематокрит > 27% спустя 1 день после начала терапии, терапия с помощью комбинации препаратов артеметер и люмефантрин и регистрация уровня в 2014–2015 гг. независимо предсказывали БППД1. Параллельно с этим, по оценкам Каплана-Мейера, риск повторных инфекций к 28 дню возрос с 8% до 14% (P = 0,005) и с 9% до 15% (P = 0,02) для артеметер-люмефантрина и артесуната-амодиахина соответственно. Средний период полураспада при бесполой паразитемии значительно увеличился с 1,1 часа до 1,3 часов в течение двух лет (P <0,0001).

Выводы: Данные свидетельствуют о снижении с течением времени паразитологического ответа на две АКТ, что может быть следствием возникновения паразитов со сниженной восприимчивостью или снижения иммунитета к инфекционным заболеваниям у этих детей.

Translated from English version into Russian by Veronika Demeshchyk, Revised by Anna Kukharchuk, through

Disminución de la capacidad de respuesta de las infecciones infantiles por *Plasmodium falciparum* a los tratamientos combinados a base de artemisinina diez años después de la implementación como antimaláricos de primera línea en Nigeria

Akintunde Sowunmi, Godwin Ntadom, Kazeem Akano, Folasade O. Ibironke, Adejumoke I. Ayede, Chimere Agomo, Onikepe A. Folarin, Grace O. Gbotosho, Christian Happi, Stephen Oguche, Henrietta U. Okafor, Martin Meremikwu, Philip Agomo, William Ogala, Ismaila Watila, Olugbenga Mokuolu, Finomo Finomo, Joy C. Ebenebe, Nma Jiya, Jose Ambe, Robinson Wammanda, George Emechebe, Wellington Oyibo, Francis Useh, Temitope Aderoyeje, Titilope M. Dokunmu, Omobolaji T. Alebiosu, Sikiru Amoo, Oluwabunmi K. Basorun, Olubunmi A. Wewe, Chukwuebuka Okafor, Odafe Akpoborie, Bayo Fatunmbi, Elsie O. Adewoye, Nnenna M. Ezeigwe y Ayoade Oduola

Resumen

Antecedentes: el desarrollo y la diseminación de la malaria por *Plasmodium falciparum* resistente a la artemisinina en la subregión del Gran Mekong ha generado un impulso para el monitoreo mundial continuo de la eficacia de las terapias combinadas basadas en artemisinina (ACTs por sus siglas en inglés). Este análisis posterior tiene como objetivo evaluar los cambios en los marcadores de respuesta al tratamiento temprano diez años después de la adopción de las ACTs como tratamiento de primera línea de la malaria por P. falciparum sin complicaciones en Nigeria.

Métodos: en 14 sitios de control en seis áreas geográficas de Nigeria, evaluamos las respuestas al tratamiento en 1341 niños menores de cinco años y adicionalmente en 360 niños menores de 16 años con malaria no complicada inscrita en ensayos aleatorios de artemeter-lumefantrina *versus* artesunato-amodiaquina en intervalos de 5 años en 2009-2010 y 2014-2015 y en intervalos de 2 años en 2009-2010 y 2012-2015, respectivamente, después de la implementación en 2005.

Resultados: la positividad del parásito asexual 1 día después del inicio del tratamiento (APPD1) aumentó de 54% a 62% y 2 días después del inicio del tratamiento de 5% a 26% en 2009-2010 a 2014-2015 (P = 0,002 y P <0,0001, respectivamente). El tiempo de eliminación del parásito aumentó significativamente de 1,6 días (intervalo de confianza [*IC*] del 95%: 1,55–1,64) a 1,9 días (*IC del 95%*: 1,9–2,0) y la relación de reducción de parásitos media geométrica 2 días después del inicio del tratamiento disminuyó significativamente de 11 000 a 4700 dentro del mismo período de tiempo (P <0,0001 para cada uno). Parasitemia de inscripción > 75 000 μl^{-1}, hematocrito > 27% 1 día de inicio del tratamiento, el tratamiento con artemeter-lumefantrina y la inscripción en 2014-2015 predijeron de forma independiente APPD1. Paralelamente a esto, el riesgo estimado con el método Kaplan-Meier de infecciones recurrentes para el día 28 aumentó de 8% a 14% (P = 0,005) y de 9% a 15% (P = 0,02) con artemeter-lumefantrina y artesunato-amodiaquina, respectivamente. La vida media de la parasitemia asexual aumentó significativamente de 1,1 horas a 1,3 horas en un periodo de dos años (P <0,0001).

Conclusiones: estos datos indican que la disminución de las respuestas parasitológicas a lo largo del tiempo para los dos ACTs podría ser debido a la aparición de parásitos con una susceptibilidad reducida o una disminución de la inmunidad a las infecciones en estos niños.

Translated from English version into Spanish by Ignacio Montalvo, Revised by Paula Hernandez, through

TRANSLATORS WITHOUT BORDERS
